# Clinical and molecular characterization of ovarian carcinoma displaying isolated lymph node relapse

**DOI:** 10.1016/j.ajog.2019.04.035

**Published:** 2019-09

**Authors:** Robert L. Hollis, Juliet Carmichael, Alison M. Meynert, Michael Churchman, Amelia Hallas-Potts, Tzyvia Rye, Melanie MacKean, Fiona Nussey, Colin A. Semple, C. Simon Herrington, Charlie Gourley

**Affiliations:** a Nicola Murray Centre for Ovarian Cancer Research, Cancer Research UK Edinburgh Centre, MRC Institute of Genetics and Molecular Medicine, University of Edinburgh, Edinburgh, United Kingdom; b MRC Human Genetics Unit, MRC Institute of Genetics and Molecular Medicine, University of Edinburgh, Edinburgh, United Kingdom; c Edinburgh Cancer Centre, Western General Hospital, Edinburgh, United Kingdom

**Keywords:** Cancer recurrence, isolated lymph node relapse, ovarian cancer, survival, tumor-infiltrating lymphocytes

## Abstract

**Background:**

Disease relapse is the primary cause of death from ovarian carcinoma. Isolated lymph node relapse is a rare pattern of ovarian carcinoma recurrence, with a reported median postrelapse survival of 2.5 to 4 years. To date, investigations have not compared isolated lymph node relapse ovarian carcinoma directly to a matched extranodal relapse cohort or performed molecular characterization of cases that subsequently experience isolated lymph node relapse.

**Objective:**

Here we seek to compare the clinical outcome, tumor-infiltrating lymphocyte burden, and frequency of known prognostic genomic events in isolated lymph node relapse ovarian carcinoma vs extranodal relapse ovarian carcinoma.

**Study Design:**

Forty-nine isolated lymph node relapse ovarian carcinoma patients were identified and matched to 49 extranodal relapse cases using the Edinburgh Ovarian Cancer Database, from which the clinical data for identified patients were retrieved. Matching criteria were disease stage, histologic subtype and grade, extent of residual disease following surgical debulking, and age at diagnosis. Clinicopathologic factors and survival data were compared between the isolated lymph node relapse and extranodal relapse cohorts. Genomic characterization of tumor material from diagnosis was performed using panel-based high-throughput sequencing and tumor-infiltrating T cell burden was assessed using immunohistochemistry for CD3+ and CD8+ cells.

**Results:**

Isolated lymph node relapse cases demonstrated significantly prolonged postrelapse survival and overall survival vs extranodal relapse upon multivariable analysis (HR^multi^ = 0.52 [0.33–0.84] and 0.51 [0.31–0.84]). Diagnostic specimens from high-grade serous ovarian carcinomas that subsequently displayed isolated lymph node relapse harbored significantly greater CD3+ and CD8+ cell infiltration compared to extranodal relapse cases (*P* = .001 and *P* = .009, Bonferroni-adjusted *P* = .003 and *P* = .019). Isolated lymph node relapse high-grade serous ovarian carcinoma cases did not show marked enrichment or depletion of cases with *BRCA1/2* mutation or *CCNE1* copy number gain when compared to their extranodal relapse counterparts (24.4% vs 19.4% and 18.2% vs 22.6%, *P* = .865 and *P* = .900).

**Conclusion:**

Isolated lymph node relapse ovarian carcinoma represents a distinct clinical entity with favorable outcome compared to extranodal relapse. There was no clear enrichment or depletion of *BRCA1/2* mutation or *CCNE1* gain in the isolated lymph node relapse ovarian carcinoma cohort compared with extranodal relapse cases, suggesting that these known prognostic genomically defined subtypes of disease do not display markedly altered propensity for isolated lymph node relapse. Diagnostic tumor material from isolated lymph node relapse patients demonstrated greater CD3+ and CD8+ cell infiltration, indicating stronger tumor engagement by T cell populations, which may contribute to the more indolent disease course of isolated lymph node relapse.

Ovarian carcinoma (OC) is the most lethal gynecologic malignancy, accounting for over 180,000 deaths per year worldwide.^1^ OC is now recognized to comprise 5 core histologic subtypes: high-grade serous (HGS), endometrioid, clear cell, low-grade serous, and mucinous OC—each displaying distinct molecular landscapes and clinical behavior.^2^ Within HGS cases, homologous recombination deficiency by virtue of *BRCA1* or *BRCA2* mutation has been associated with favorable outcome, greater sensitivity to platinum-based chemotherapy, and marked benefit from poly (ADP-ribose) polymerase inhibitors.^3^, ^4^, ^5^, ^6^ Conversely, *CCNE1* copy number gain has been associated with chemoresistance and poorer survival in this group.^3^, ^7^

**AJOG at a Glance**

**Why was this study conducted?**

A number of investigators have reported a relatively indolent disease course in ovarian carcinoma patients experiencing isolated lymph node relapse. However, none have systematically compared these to extranodal relapse or performed molecular characterization of patients who go on to experience this distinct pattern of recurrence.

**Key findings**

Isolated lymph node relapse patients demonstrated significantly prolonged overall and postrelapse survival compared with extranodal relapse cases. Isolated lymph node relapse cases demonstrated greater tumor-infiltrating lymphocyte burden at diagnosis, but did not demonstrate significant enrichment or depletion of *BRCA1/2* mutation or gain of *CCNE1*, both known to be prognostic in ovarian carcinoma.

**What does this add to what is known?**

This is the first report demonstrating significantly improved clinical outcome in isolated lymph node relapse ovarian carcinoma when compared directly with extranodal relapse, and represents the first study to perform molecular characterization of patients who go on to experience isolated lymph node relapse.

Though patients in most OC cases—particularly HGS OCs—are typically sensitive to chemotherapy in the first-line setting, the majority of patients will experience disease relapse, which acquires resistance to chemotherapy.^8^, ^9^ The most common sites of recurrence are the pelvis and peritoneum.^10^ Involvement of lymph nodes (LNs) at relapse is common; however, recurrence confined solely to LNs is a rare event, accounting for ≤5% of relapsed OCs.^11^, ^12^ These isolated LN relapse (ILNR) cases have been described as a unique clinical disease entity and are thought to experience a relatively indolent disease course, with a reported median postrelapse survival (PRS) and overall survival (OS) of around 2.5 to 4 years and >5 years, respectively.^11^, ^12^, ^13^, ^14^, ^15^, ^16^, ^17^, ^18^

A number of previous studies have reported on the clinical outcome of apparent ILNR OC (summarized in Table 1).^11^, ^12^, ^13^, ^14^, ^15^, ^16^, ^17^, ^18^ Many of these studies have reported only a small number of cases,^11^, ^13^, ^17^, ^18^ with a minority reporting larger numbers identified from multiple centers.^14^, ^16^ To our knowledge, none of these studies have compared outcome directly to a matched extranodal relapse (ENR) cohort. Furthermore, they have not performed contemporary histologic subtyping or molecular characterization so as to identify potential subgroups of disease with a propensity to experience this distinct pattern of disease relapse.

**Table 1:** Previous reports of isolated lymph node relapse ovarian carcinoma

ILNR cases	N	Ferrero^16^	Tu^15^	Gadducci^14^	Fotiou^18^	Legge^12^	Santillan^17^	Blanchard^11^	Uzan^13^	Summary
		73	38	69	21	32	25	27	12	Few reports of ≥40 cases
Age at diagnosis	Years	Median 54	24 (63%) >50; 14 (37%) ≤50	Median 58	Mean 50	Median 60	Mean 58	Mean 59	Median 51	Largely unremarkable compared with unselected OC cohorts
	Range	29–73		34–78	36–67	45–76	41–82	41-85	42–71	
Stage at diagnosis	I	14 (19%)	0	11 (16%)	3 (14%)	0	2 (8%)	4 (15%)	5 (42%)	
	II	4 (6%)	15 (39%)	6 (9%)	3 (14%)	1 (3%)	5 (20%)	5 (18%)	1 (8%)	
	III	51 (70%)	23 (61%)	46 (67%)	14 (67%)	29 (91%)	15 (60%)	15 (56%)	6 (50%)	
	IV	4 (6%)	0	6 (9%)	1 (5%)	2 (6%)	3 (12%)	3 (11%)	0	
RD following first-line debulking	0 cm / <0.5 cm	57 (78%)	17 (45%)	22 (32%)	8 (38%)	14 (44%)	18 (72%)	NA	7 (58%)	
	≤1 cm	10 (14%)	10 (26%)	11 (16%)	7 (33%)	6 (19%)	5 (20%)	NA	4 (33%)	
	<2 cm	4 (6%)	11 (29%)	36 (52%)	4 (19%)		0	NA		
	>2 cm	2 (3%)			2 (10%)	12 (38%)	2 (8%)	NA	1 (8%)	
Grade at diagnosis	I	4 (6%)	7 (18%)	3 (4%)	0	9 (32%)	25 (100%) high grade	NA	NA	
	II	5 (7%)	14 (37%)	13 (19%)	8 (38%)					
	III	64 (88%)	17 (45%)	54 (78%)	13 (62%)	19 (68%)				
	NA	-	-	-	-	4	-			
Reported histologic subtype at diagnosis	Serous	53 (73%)	19 (50%)	52 (75%)	16 (76%)	26 (81%)	19 (76%)	17 (62%)^a^	8 (67%)	Predominantly serous / HGS cases, as with unselected OC cohorts
	Endometrioid	11 (15%)	9 (24%)	12 (17%)	5 (24%)	2 (6%)	2 (8%)	3 (11%)	3 (25%)	
	Clear cell	0	0	1 (1%)		0	0	0	0	
	Mucinous	1 (1%)	1 (3%)	0		1 (3%)	0	3 (11%)	0	
	Other	8 (11%)	9 (24%)	4 (6%)		3 (9%)	4 (16%)	4 (15%)	1 (8%)	
DFI / time to ILNR^b^	Median months	18	18	44 (62%) >12 months	21	17.5	16	26 months from diagnosis	21	Median 1.5–2 years DFI
	Range	6–192	9-96		8–156	1–134	6–40	1–159	6–72	
ILNR site(s)	Para-aortic only	37 (51%)	10 (26%)	23 (33%)	8 (38%)	14 (44%)	15 (60%)	9 (33%) retro. alone, 6(22%) retro. + other. supraclavicular, mediastinal, iliac, and inguinal involvement in 7 (26%), 4 (15%), 4 (15%), and 3 (11%) cases	5 (42%)	Most commonly involves pelvic and/or para-aortic sites
	Pelvic only	21 (29%)	15 (39%)	12 (17%)	4 (19%)	1 (3%)	3 (12%)		4 (33%)	
	Para-aortic & pelvic	9 (12%)	7 (18%)	6 (9%)	4 (19%)	9 (28%)	1 (4%)		1 (8%)	
	Inguinal only	3 (4%)	2 (5%)	12 (17%)	4 (19%)	2 (6%)	5 (20%)		1 (8%)	
	Other combinations	3 (4%)	4 (11%)	16 (23%)	1 (5%)	6 (19%)	1 (4%)		1 (8%)	
ILNR pattern	Single region	61 (84%)	27 (71%)	47 (77%)	17 (81%)	20 (63%)	24 (96%)	17 (63%)	10 (83%)	Most commonly localized to a single region
	Multiregion	12 (16%)	11 (29%)	14 (23%)	4 (19%)	9 (28%)	1 (4%)	10 (37%)	2 (17%)	
	NA	-	-	8	-	3	-	-	-	
PRS	Median months	5-y PRS 64%; 5-y OS ∼80%	5-y PRS 66.5%	32.1	47	37	37	26	5-y PRS 71%	Median 2–4 years
OS	Median months			62.9	66	109	61	68		Median >5 years
Surgery for ILNR	Yes	73 (100%)	19 (50%)	24 (35%)	21 (100%)	12 (38%)	25 (100%)	8 (30%)	12 (100%)	Heterogeneous management, typically involving chemotherapy
	No	0	19 (50%)	45 (65%)	0	20 (63%)	0	19 (70%)	0	
ILNR intervention: regime	Chemo alone	0	5 (13%)	44 (64%)	0	19 (59%)	0	8 (30%)	0	
	Surgery alone	3 (4%)	0	1 (1%)	0	1 (3%)	2 (8%)	2 (7%)	0	
	Surgery-chemo combination	70 (96%)	19 (50%)	22 (32%)	17 (81%)	11 (34%)	15 (60%)	5 (19%)	10 (83%)	
	Radio alone	0	0	1 (1%)	0	0	0	2 (7%)	0	
	No intervention	0	0	0	0	1 (3%)	0	7 (26%)	0	
	Other	0	14 (37%)	1 (1%)	4 (19%)	0	8 (32%)	3 (11%)	2 (17%)	

*Chemo*, chemotherapy; *DFI*, disease-free interval; *ENR*, extranodal relapse; *ILNR*, isolated lymph node relapse; *NA*, not available; *OS*, overall survival; *PRS*, postrelapse survival; *radio*, radiotherapy; *RD*, residual disease; *retro*, retroperitoneal.

*Hollis et al. Clinical and molecular characterization of ILNR ovarian carcinoma. Am J Obstet Gynecol 2019*.

a Includes 5 cases described as papillary

b From end of first-line chemotherapy.

Here, we report clinical and molecular characterization of a matched ILNR and ENR cohort with contemporary pathology review to compare the clinical outcome and molecular landscape of ILNR and ENR OC.

## Materials and Methods

### Isolated lymph node relapse patient identification

**Supplementary Figure S1:**
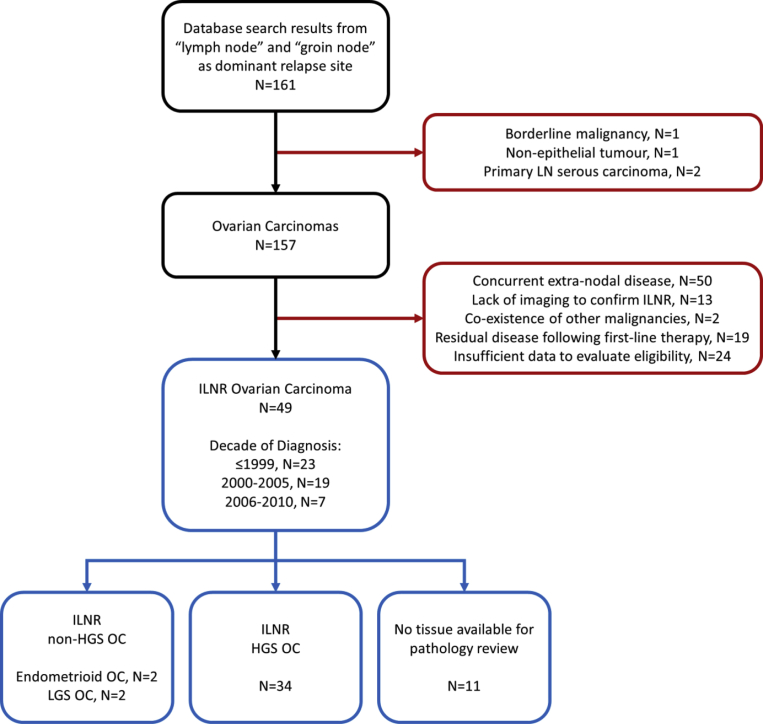
Isolated lymph node relapse cohort identification from the Edinburgh Ovarian Cancer Database *Hollis et al. Clinical and molecular characterization of ILNR ovarian carcinoma. Am J Obstet Gynecol 2019*.

ILNR OC cases were identified from the Edinburgh Ovarian Cancer Database (Appendix: Supplementary Figure S1), wherein the clinical variables, treatment details, and follow-up data of OC patients treated within the Edinburgh Cancer Centre are collected prospectively as part of routine care. Potential ILNR cases were identified using the search terms “lymph node” or “groin node” as the dominant site of relapse, yielding 161 results. Nonepithelial tumors (n = 1), tumors of borderline malignancy (n = 1), and primary LN serous carcinomas (n = 2) were excluded. Patients with concurrent extranodal disease (n = 50), lack of cross-sectional imaging to confirm sole ILNR (n = 13), or coexistence of other malignancies leading to uncertain origin of LN disease (n = 2) were excluded. Patients with residual disease (RD) after completion of first-line treatment (n = 19) or insufficient clinical data for eligibility assessment (n = 24) were also excluded, leaving 49 ILNR cases.

### Matching of isolated lymph node relapse to extranodal relapse

ILNR cases were electronically matched to ENR cases with complete response to first-line therapy using the Edinburgh Ovarian Cancer Database. Matching criteria were as follows: (1) diagnostic histologic subtype and grade, (2) stage at diagnosis, (3) extent of RD following debulking surgery, and (4) closest age at diagnosis following matching of criteria 1–3. Criteria were relaxed to facilitate matching of all ILNR cases as detailed in Supplementary Table S1 (Appendix).

### Ethical approval and tissue collection

Clinical research access and ethical approval for correlation of molecular data to clinicopathologic features and clinical outcome in OC was obtained via NHS Lothian Research and Development (reference ID 2007/W/ON/29). Ethical approval for the use of human tumor material in translational research was obtained from South East Scotland Human Annotated Bioresource (Lothian NRS Bioresource Ethics Reference 15/ES/0094-SR831). Tumor material was available for 75.5% (74 of 98) of cases (77.6%, 38 of 49 ILNR and 73.5%, 36 of 49 ENR).

### Histologic subtyping of ovarian carcinomas

Contemporary pathology review of ILNR and matched ENR cases was performed by an expert gynecologic pathologist (C.S.H.). Where appropriate (n = 9), immunohistochemistry (IHC) for WT1 and p53 was performed to aid histologic subtyping.^19^ WT1 IHC was performed using 1:1000 dilutions of antibody M3561 clone 6F-H2 (Dako, Agilent Technologies, Santa Clara, CA). p53 staining was performed using 1:50 dilutions of antibody M7001 clone DO-7 (Dako, Agilent Technologies). Both stains were performed using the Leica Bond III Autostainer (Leica Biosystems, Milton Keynes, UK).

### Nucleic acid isolation

Up to 10 10-μm formalin-fixed paraffin-embedded sections, macrodissected using marked hematoxylin–eosin-stained slides as a guide to enrich for tumor purity (Appendix: Supplementary Table S2), were used for DNA extraction. DNA was extracted using the QIAamp DNA FFPE Tissue Kit and Deparaffinization Solution (Qiagen, Venlo, the Netherlands).

### Panel-based sequencing of *BRCA* and non-*BRCA* homologous recombination deficiency genes

High-throughput sequencing was performed using an 83-gene custom Integrated DNA Technologies gene capture panel with unique molecular indices, as described in the Appendix. Gene targets, centered around the homologous recombination DNA repair pathway, are detailed in Supplementary Table S3 (Appendix). The median per-sample mean target coverage achieved was 386X.

### Assessment of *CCNE1* copy number

Copy number variants in *CCNE1* were characterized by TaqMan Genotyping qPCR Copy Number Assays (Applied Biosystems, Thermo Fisher Scientific, Waltham, MA), as detailed in the Appendix.

### Assessment of tumor-infiltrating lymphocyte density

**Supplementary Figure S2:**
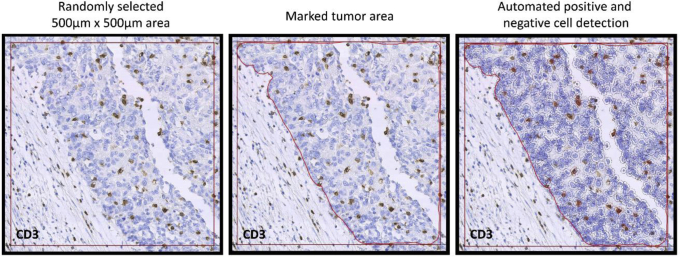
Automated marker-positive cell quantification by QuPath *Hollis et al. Clinical and molecular characterization of ILNR ovarian carcinoma. Am J Obstet Gynecol 2019*.

Tumor-infiltrating lymphocytes (TILs) were assessed using 4-μm FFPE sections of diagnostic tumor material from first-line cytoreductive surgery. IHC for CD3 and CD8 was performed using Bond ready-to-use CD8-4B11 and CD3-LN10 antibodies (Leica Biosystems) on the Leica Bond III Autostainer. Human tonsil was used as a positive control for both markers. Stained slides were digitized and marker-positive cells were quantified using QuPath^20^ in 8 randomly selected tumor-containing 500 × 500-μm fields per sample. Tumor area was marked as a region of interest (Appendix: Supplementary Figure S2) and marker-positive cells were quantified using the positive cell detection protocol as a percentage of the total cell number demonstrating marker positivity.

TIL scoring validation was performed by manual counting of marker-positive cells by 2 human observers (R.L.H. and A.H.P.), in a randomly selected validation cohort representing 15% of samples for each marker. The correlation of marker-positive cell counts (observer 1 vs observer 2 vs QuPath) demonstrated excellent agreement for both markers (Spearman’s rho > 0.95, *P* < .0001 for all comparisons).

### Statistical analyses

Statistical analyses were performed using R version 3.5.1 (R Foundation, Vienna, Austria). Disease-free interval (DFI) was calculated as time from end of first-line chemotherapy to disease recurrence. Comparisons of OS and PRS were conducted using Cox proportional hazards regression models within the Survival R package^21^ and presented as hazard ratios (HRs) alongside their 95% confidence intervals (CIs). Frequency comparisons were made using the χ^2^ test and Fisher exact test as appropriate. Comparisons of TIL density were made using the Mann–Whitney *U* test. Analyses were adjusted for multiplicity of testing using the Bonferroni correction, where specified.

## Results

### Cohort characteristics

Demographics of the ILNR and ENR cohorts are summarized in Table 2. There was no significant difference in age at diagnosis, RD following primary surgical debulking, histology or grade of disease at diagnosis, or disease stage at diagnosis between the ILNR and ENR groups. These data indicate good fidelity of the ILNR–ENR matching process. Patterns of ILNR are described in Table 3.

**Table 2:** Demographics of isolated lymph node relapse and extranodal relapse ovarian carcinoma cohorts

Factor	Class	ILNR, n = 49		ENR, n = 49		ILNR vs ENR
		N	%/range	N	%/range	*P* value
Stage at diagnosis	I	5	10.6	5	10.2	1.000^a^
	II	10	21.3	11	22.4	
	III	27	57.4	28	57.1	
	IV	5	10.6	5	10.2	
	NA	2		0		
Histology at diagnosis	Serous	25	51.0	33	67.3	.502^b^
	Endometrioid	12	24.5	11	22.5	
	Clear cell	1	2.0	1	2.0	
	Mixed histology	8	16.3	4	8.2	
	Unclassified adenocarcinoma	3	6.1	0	0.0	
Grade at diagnosis	I	0	0.0	1	2.0	1.000^c^
	II	6	13.0	6	12.2	
	III	40	87.0	42	75.7	
	NA	3		0		
Contemporary histologic classification	HGS	34	89.5	31	86.1	.733^d^
	Endometrioid	2	5.3	3	8.3	
	LGS	2	5.3	1	2.8	
	Mixed HGS / endometrioid	0	0.0	1∗	2.8	
	No specimen available	11		13		
Surgical debulking status	RD <2 cm	34	75.6	33	70.2	.733^e^
	RD 2–5 cm	7	15.6	8	17.0	
	RD ≥5 cm	4	8.9	6	12.8	
	NA	4		2		
First-line chemotherapy	Platinum	21	42.9	17	34.7	.693^f^
	Platinum combination	25	51.0	28	57.1	
	Other	3	6.1	4	8.2	
Neoadjuvant first-line chemotherapy	Yes	2	4.1	1	2.0	1.000^g^
	No	47	95.9	48	98.0	
Year of diagnosis	≤1999	23	46.9	21	42.9	.667^f^
	2000–2005	19	38.8	23	46.9	
	2006–2010	7	14.3	5	10.2	
Age at diagnosis	Median years	61	41-80	62	41-80	.339^h^
Specimen from diagnosis	Primary site	33	91.7	29	80.6	.307^i^
	Omentum	2	5.6	6	16.7	
	Other	1	2.8	1	2.8	
	NA	2		0		
	No specimen available	11		13		

*ENR*, extranodal relapse; *ILNR*, isolated lymph node relapse; *NA*, not available; *RD*, residual disease.

*Hollis et al. Clinical and molecular characterization of ILNR ovarian carcinoma. Am J Obstet Gynecol 2019*.

a χ^2^ test, stage I/II vs stage III/IV

b χ^2^ test, serous/mixed vs other

c χ^2^ test, grade I/II vs grade III

d Fisher exact test, high-grade serous vs non-high-grade serous

e χ^2^ test, RD <2 cm vs ≥2 cm

f χ^2^ test

g Fisher exact test

h Welch 2-sample *t* test

i Fisher exact test, primary site vs omentum/other

∗ This tumor had 2 morphologically distinct components with different immunophenotypes.

**Table 3:** Patterns of isolated lymph node relapse ovarian carcinoma

	Cases (n)	Proportion of cases (%)
ILNR pattern		
Single-site	22	44.9
Multiregional		
2	17	34.7
3	8	16.3
4	2	4.1
ILNR sites		
Para-aortic only	16	32.7
Pelvic only	4	8.2
Inguinal only	2	4.1
Pelvic & para-aortic	6	12.2
Supraclavicular and other sites	6	12.2
Pelvic, para-aortic, and other(s)	6	12.2
Other combinations	9	18.4

*ILNR*, isolated lymph node relapse.

*Hollis et al. Clinical and molecular characterization of ILNR ovarian carcinoma. Am J Obstet Gynecol 2019*.

### Clinical outcome in isolated lymph node relapse vs extranodal relapse

**Figure 1:**
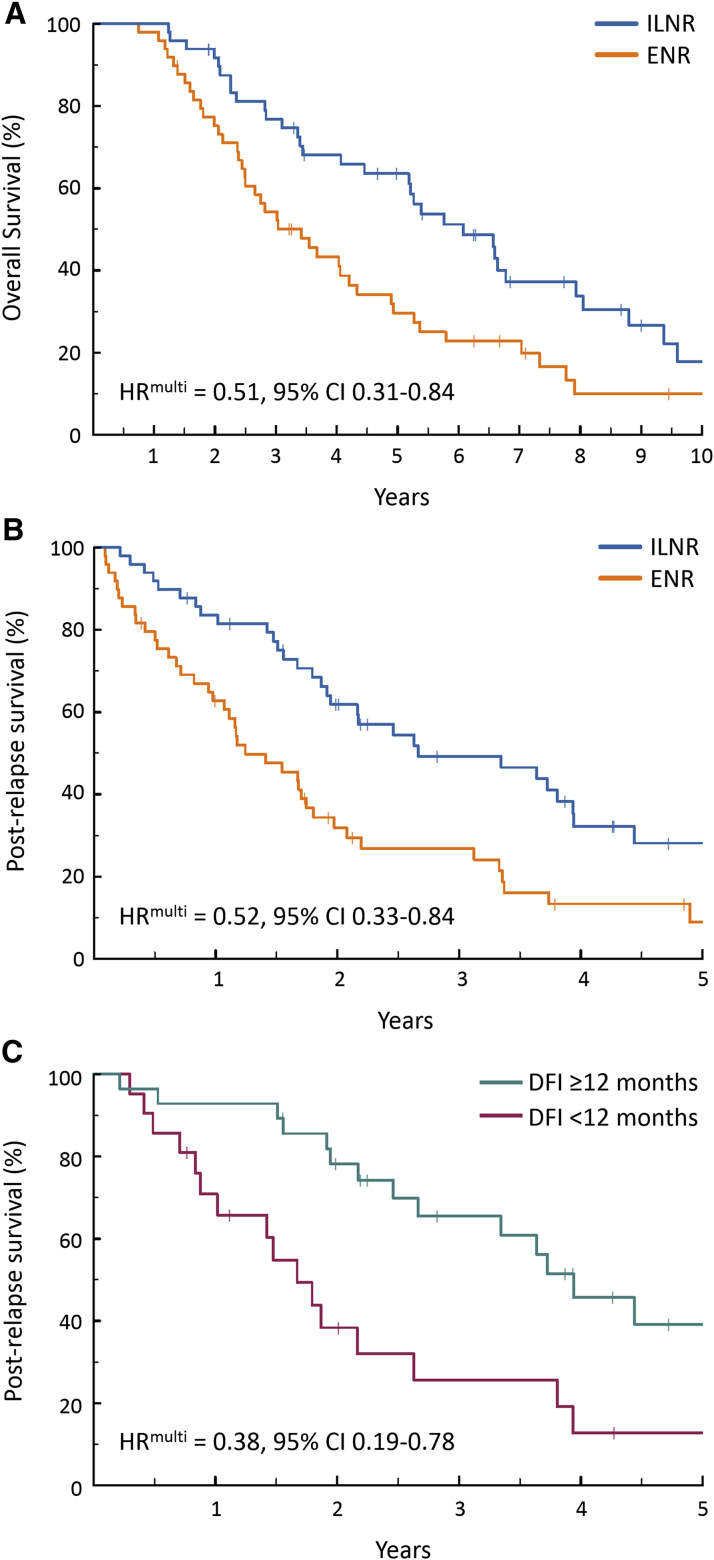
Clinical outcome of isolated lymph node relapse OC **A,** Overall survival in ILNR vs ENR OC. **B,** Postrelapse survival in ILNR vs ENR OC. **C,** Postrelapse survival in ILNR OC by DFI length. *CI*, confidence interval; *DFI*, disease-free interval; *ENR*, extranodal relapse; *HR*, hazard ratio; *ILNR*, isolated lymph node relapse; *OC*, ovarian carcinoma. *Hollis et al. Clinical and molecular characterization of ILNR ovarian carcinoma. Am J Obstet Gynecol 2019*.

ILNR patients displayed prolonged OS and PRS compared with the ENR cohort (HR = 0.55 [0.34–0.87], *P* = .011 and HR = 0.50 [0.31–0.80], *P* = .004) (Figure 1, A and Figure 1, B). The median OS and PRS in the ILNR cohort was 72.9 (95% CI 62.2–96.5) and 32.0 (95% CI 23.3–53.3) months, compared with 41.1 (95% CI 30.0–58.8) and 14.9 (95% CI 12.9–23.7) months in the ENR cohort. The length of the DFI prior to ILNR or ENR was not significantly different between the 2 cohorts (HR = 0.86 [0.60–1.29], *P* = .473).

Multivariable analysis for OS accounting for extent of RD following primary debulking, FIGO stage, and age at diagnosis identified significantly prolonged OS in the ILNR cohort (HR^multi^ = 0.51 [0.31–0.84], *P* = .008) (Appendix: Supplementary Table S4). Multivariable analysis of PRS, accounting for DFI and age, identified prolonged PRS in ILNR cases (HR^multi^ = 0.52 [0.33–0.84], *P* = .007) (Appendix: Supplementary Table S5).

Significantly prolonged OS (HR^multi^ for OS = 0.53 [0.29–0.99], *P* = .046) and PRS (HR^multi^ for PRS = 0.54 [0.31–0.96], *P* = .037) was demonstrated for ILNR OC when HGS cases were considered specifically (34 ILNR HGS OCs, 31 ENR HGS OCs).

### Longer disease-free interval is associated with prolonged postrelapse survival in isolated lymph node relapse ovarian carcinoma

The importance of DFI on clinical outcome in ILNR OC remains controversial, with some authors reporting no association between DFI length and PRS or OS in this setting^11^, ^16^, ^18^ and others reporting significant associations.^12^, ^14^, ^15^ Within the ILNR cohort, DFI ≥12 months was associated with markedly prolonged PRS when accounting for patient age (HR^multi^ = 0.38 [0.19–0.78], *P* = .008), with median PRS of 47.3 months vs 20.1 months in those with DFI ≥12 months and DFI <12 months, respectively (Figure 1, C).

### Impact of isolated lymph node relapse pattern on outcome

There was no clear differential PRS between multiregion ILNR and single-region ILNR (2 regions vs single-site HR = 1.06 [0.49–2.30], *P* = .890; ≥3 sites vs single-site HR = 0.94 [0.36–1.43], *P* = .898).

**Supplementary Figure S3:**
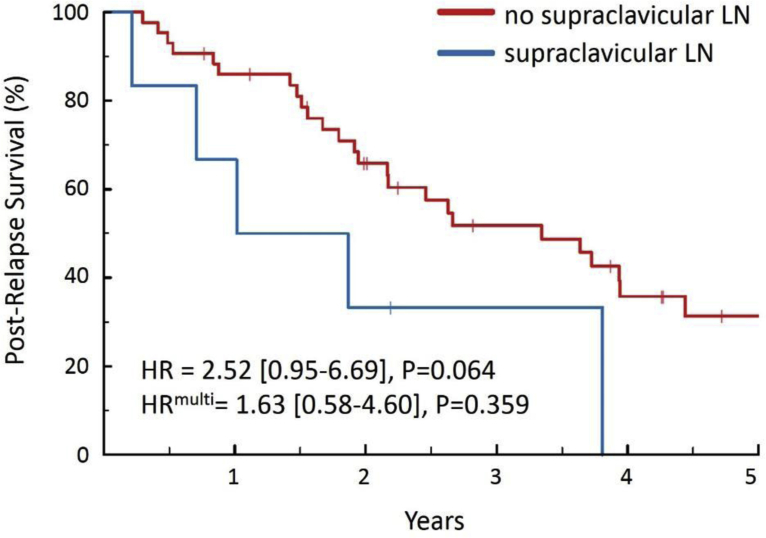
Postrelapse survival of isolated lymph node relapse ovarian carcinoma with and without supraclavicular lymph node involvement *Hollis et al. Clinical and molecular characterization of ILNR ovarian carcinoma. Am J Obstet Gynecol 2019*.

Six ILNR cases (12.2%) involved supraclavicular LN sites. Although these cases demonstrated an apparent trend for inferior PRS (HR = 2.52 [0.95–6.69], *P* = .064) (Appendix: Supplementary Figure S3), there was no significant difference after accounting for DFI and age (HR^multi^ = 1.63 [0.58–4.60], *P* = .359). Other specific LN sites were not associated with apparent differential PRS (Appendix: Supplementary Table S6).

### Molecular landscape of isolated lymph node relapse high-grade serous ovarian carcinoma

**Figure 2:**
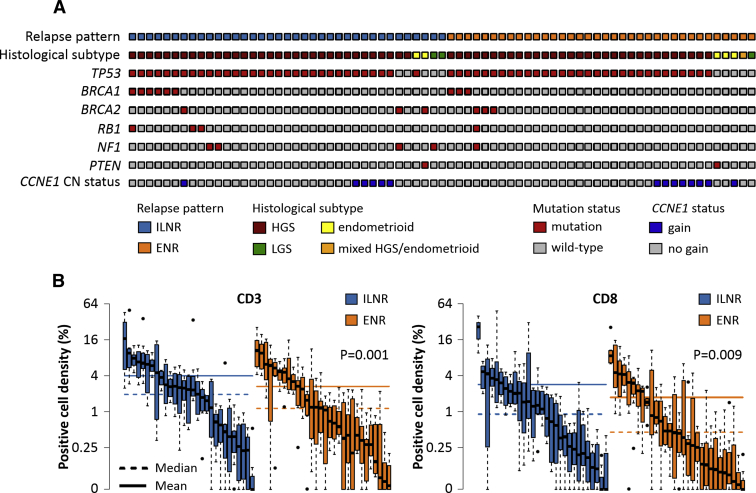
Molecular landscape of isolated lymph node relapse and extranodal relapse ovarian carcinoma **A,** Genomic events in ILNR and ENR cases. **B,** Tumor-infiltrating lymphocyte burden of ILNR and ENR HGS ovarian carcinomas. *ENR*, extranodal relapse; *HGS*, high-grade serous; *ILNR*, isolated lymph node relapse; *LGS*, low-grade serous. *Hollis et al. Clinical and molecular characterization of ILNR ovarian carcinoma. Am J Obstet Gynecol 2019*.

Sixty-four HGS OC cases (33 ILNR, 31 ENR) were successfully characterized for HR gene mutations and *CCNE1* copy number. Frequencies of genomic abnormalities are outlined in Figure 2, A and Supplementary Table S3. Within HGS OC cases, there was no significant difference in the rate of *CCNE1* copy number gain (18.2%, 6/33 vs 22.6%, 7/31, *P* = .900) or *BRCA1/2* mutation (24.4%, 8/33 vs 19.4%, 6/31, *P* = .865) between the ILNR and ENR cohorts (Figure 2, A).

The CD3+ and CD8+ TIL burden was greater in diagnostic tumor specimens from HGS OC patients who went on to experience ILNR when compared with their ENR counterparts (median CD3+ cell density 1.94% vs 1.13%, *P* = .001 and median CD8+ cell density 0.90% vs 0.45%, *P* = .009; Bonferroni-adjusted *P* = .003 and *P* = .019) (Figure 2, B).

## Comment

### Principal findings

The principal findings of this study are as follows: (1) ILNR represents a distinct pattern of OC relapse with prolonged survival vs ENR cases; (2) longer DFI prior to ILNR is associated with prolonged PRS in ILNR; (3) ILNR OC do not demonstrate significantly differential composition of known genomic subtypes associated with prognosis, namely *BRCA1/2* mutation or gain of *CCNE1*; (4) cases that go on to experience ILNR demonstrate greater TIL burden at diagnosis compared with ENR cases.

### Study strengths and limitations

A key strength of this study is the direct comparison of ILNR OC with matched ENR cases: a number of studies have reported ILNR as a distinct pattern of OC relapse with a relatively indolent disease course but have not systematically compared ILNR cases directly to a matched ENR cohort.^11^, ^12^, ^13^, ^14^, ^15^, ^16^, ^17^, ^18^ Moreover, these studies did not perform pathology review of identified cases, precluding the ability to characterize ILNR outcome in the context of contemporary OC histotypes, which are now known to display markedly differential clinical outcome.^22^ Critically, we characterize ILNR OC following contemporary histologic subtyping to facilitate investigation of ILNR in a histotype-specific manner.

The majority of previous studies investigating ILNR have identified fewer than 20 OC cases of serous histology that go on to experience this rare relapse pattern; moreover, previous reports have not performed molecular characterization of OC cases that demonstrate ILNR.^11^, ^12^, ^13^, ^14^, ^15^, ^16^, ^17^, ^18^ We identified 49 ILNR OC patients treated within the Edinburgh Cancer Centre, including 34 cases reviewed as HGS OC. This study represents the largest ILNR OC series from a single center and the only report investigating the molecular landscape of ILNR OC to date.

Though this study does represent one of the largest reported ILNR OC cohorts, case numbers were still restricted owing to the rarity of ILNR OC. In particular, power to detect differential outcome between distinct patterns of ILNR was limited, and we could not perform meaningful analysis comparing rates of rare genomic events present in both ILNR and ENR cohorts, including mutational events in *RB1*, *NF1*, and *PTEN*, as well as gene-specific analysis of *BRCA1* and *BRCA2*. Other limitations of this study include heterogeneous treatment of OC patients across the time period in which these cases were diagnosed, though diagnosis periods were comparable between the ILNR and ENR cohorts (Table 2).

### Clinical outcome in isolated lymph node relapse ovarian carcinoma

The median PRS and OS of ILNR cases was approximately 2.7 and 6 years, consistent with previous reports of ILNR OC.^11^, ^12^, ^13^, ^14^, ^15^, ^16^, ^17^, ^18^ ILNR cases displayed significantly prolonged OS and PRS compared to their ENR counterparts upon multivariable analysis (HR^multi^ = 0.51 and 0.52 for OS and PRS). Critically, this difference was maintained in a histotype-specific analysis of HGS cases, which account for the majority of OCs. To our knowledge, this is the first report directly demonstrating a significant difference in outcome between ILNR and ENR OC.

Only half of the reports investigating the impact of DFI length on ILNR outcome to date have identified associations with OS or PRS.^12^, ^14^, ^15^ Here, we demonstrate that DFI ≥12 months is associated with a substantial PRS benefit (median PRS approximately 3.9 vs 1.7 years), largely reflective of established associations in unselected OC cases.^23^ Although this contradicts reports from some investigators,^11^, ^16^, ^18^ 2 of these studies reported specifically in the context of ILNR undergoing secondary debulking^16^, ^18^ and the other compared cases using a cut-off DFI of 24 months, rather than 12 months as described here,^11^ potentially explaining this discrepancy. Notably, the intervals considered in our study are akin to those used clinically to define platinum sensitivity in unselected relapsed OC.^23^

We show no significant difference in clinical outcome between patients with ILNR at multiple sites vs those with single-site ILNR, or between distinct patterns of ILNR. Although univariable analysis suggested that supraclavicular LN involvement may confer inferior PRS, this trend was not apparent when accounting for DFI and patient age, suggesting that this is not a genuine phenomenon of supraclavicular ILNR. Notably, the number of patients with supraclavicular LN involvement was low (n = 6). Together, these data support the consideration of ILNR OC as a single disease entity, regardless of the number and location of involved sites.

### The genomic landscape of isolated lymph node relapse ovarian carcinoma

Until now, the molecular landscape of ILNR has been completely uncharacterized. It has therefore been unclear as to whether OC cases that go on to experience ILNR demonstrate enrichment of tumors belonging to known favorable genomic subgroups. Within unselected cohorts of HGS OC, inactivation of *BRCA1* or *BRCA2* has been associated with favorable outcome,^3^, ^4^ while copy number gain of *CCNE1* has been associated with poor survival and chemoresistance.^3^, ^7^ Genomic characterization of this cohort did not identify significant depletion or enrichment of these molecular events in ILNR HGS OC cases versus their ENR counterparts. These data suggest that the survival benefit of ILNR OC is not underpinned by large-scale enrichment for *BRCA1/2*-mutant cases with favorable prognosis or absence of *CCNE1*-gained cases that have poorer prognosis, and suggest that these genomic subgroups do not display markedly differential propensity for ILNR.

### Greater tumor-infiltrating lymphocyte burden at diagnosis in patients who subsequently experience isolated lymph node relapse

Intriguingly, assessment of the CD3+ and CD8+ cell burden in ILNR and ENR tumor material—reflective of whole T cell and cytotoxic T cell populations—uncovered significantly greater TIL burden in diagnostic tissue from patients who subsequently experienced ILNR (2-fold enrichment for CD8+ cells, approximately 1.7-fold enrichment for CD3+ cells). These data suggest that active engagement of the immune system at diagnosis impacts upon the nature of disease at relapse, and that immune-mediated control of cancer cells may contribute to the indolent disease course of ILNR OC. Indeed, these data may well be of interest in relation to the use of immune-directed therapies in cancer treatment.^24^, ^25^ However, though many ILNR cases displayed high TIL burden, some cases demonstrated relatively low levels of TILs, alluding to mechanisms beyond effective T-cell engagement at diagnosis underpinning some ILNR cases.

### Conclusion

Collectively, the data presented here—supported by previous descriptions of apparent ILNR in the literature—demonstrate that ILNR represents a distinct pattern of OC with favorable clinical outcome when compared with ENR. Patients that go on to experience ILNR harbor greater TIL burden at diagnosis, but they do not show marked enrichment or depletion of known genomic subgroups associated with differential outcome.

## Figures and Tables

**Supplementary Table S1:** Tolerances for electronic matching of extranodal relapse to isolated lymph node relapse

Feature	ILNR OC case documented diagnostic characteristic	Matched ENR OC case documented diagnostic characteristic	Patients
Stage at diagnosis	Inadequate information to stage	Stage II, n = 1	3
		Stage IIIC, n = 1	
	Stage IIB	Stage IIA, n = 1	
Documented histology at diagnosis	Adenocarcinoma	Serous papillary, n = 3	9
	Endometrioid	Mixed serous/endometrioid, n = 1	
	Mixed histology	Serous papillary, n = 5	
Grade at diagnosis	NA	Grade I, n = 1	3
		Grade III, n = 2	
RD following debulking	<2 cm	NA, n = 1	4
	NA	2–5 cm, n = 1	
		>5 cm, n = 2	

The matching criteria were relaxed for 2 fields for 2 patients and 3 fields for 1 patient, as follows: 1 x stage IIB grade III mixed histology carcinoma with RD <2 cm matched to stage IIA grade III serous carcinoma with RD <2 cm; 1 x stage IV unclassified adenocarcinoma of unknown grade and RD <2 cm matched to stage IV grade III serous carcinoma with RD <2 cm; 1 x stage IV unclassified adenocarcinoma of unknown grade and RD not available matched to stage IV grade III serous carcinoma with RD >5 cm.

*ENR*, extranodal relapse; *ILNR*, isolated lymph node relapse; *NA*, not available; *OC*, ovarian carcinoma; *RD*, residual disease.

*Hollis et al. Clinical and molecular characterization of ILNR ovarian carcinoma. Am J Obstet Gynecol 2019*.

**Supplementary Table S2:** Cellularity of specimens used for DNA extraction

Tumor cellularity of macrodissected area	Number (%) of cases
<20%	3 (4.1%)
20–39%	7 (9.5%)
40–59%	9 (12.2%)
60–79%	26 (35.1%)
≥80%	29 (39.2%)

*Hollis et al. Clinical and molecular characterization of ILNR ovarian carcinoma. Am J Obstet Gynecol 2019*.

**Supplementary Table S3:** Frequency of patients with detrimental mutations in genes sequenced on Integrated DNA Technologies gene capture high throughput sequencing panel

Gene	ILNR OC with mutation	ENR OC with mutation
*ABCB1*	1	0
*ARID1A*	1	2
*ATM*	2	0
*ATR*	1	0
*BRCA1*	6	3
*BRCA2*	3	3
*CTNNB1*	1	1
*FANCC*	0	1
*KRAS*	1	1
*MSH2*	1	1
*NF1*	4	1
*PIK3CA*	2	1
*PRKDC*	1	0
*PTEN*	1	1
*RB1*	3	1
*SLX4*	0	1
*TP53*	32	31
Genes with no detected mutations: *ATRX, BAP1, BARD1, BCL2L1, BLM, BRAF, BRIP1, C11orf65, CCNE1, CDK12, CHD4, CHEK1, CHEK2, EGFR, EMSY, ERBB2, ERCC4, EZH2, FANCA, FANCB, FANCD2, FANCE, FANCF, FANCG, FANCI, FANCL, FANCM, GNAS, KIT, MAD2L2, MDM2, MLH1, MRE11, MSH6, MUS81, MUTYH, NBN, NDUFB2, NF2, NRAS, PALB2, PARP1, PARP2, PAXIP1, PDGFRA, PER3, PMS2, PPP2R1A, PPP2R2A, RAD50, RAD51, RAD51B, RAD51C, RAD54L, RNASEH2A, RNASEH2B, RNASEH2C, RPA1, RUNDC3B, SHFM1, SLC25A40, SLFN11, TOE1, TP53BP1, UBE2T, VRK2*		

*ENR*, extranodal relapse; *ILNR*, isolated lymph node relapse; *OC*, ovarian carcinoma.

*Hollis et al. Clinical and molecular characterization of ILNR ovarian carcinoma. Am J Obstet Gynecol 2019*.

**Supplementary Table S4:** Multivariable analysis for overall survival in isolated lymph node relapse vs extranodal relapse in ovarian carcinoma

Factor	Class	N	HR^multi^	Lower 95% CI	Upper 95% CI	*P*
Relapse type	ILNR	49	0.51	0.31	0.84	.008
	ENR	49	Ref	Ref	Ref	Ref
Stage at diagnosis	Early (I/II)	31	0.41	0.17	1.02	.055
	III	55	0.45	0.19	1.03	.060
	IV	10	Ref	Ref	Ref	Ref
	NA	2	-	-	-	-
Surgical debulking status	RD <2 cm	67	0.60	0.32	1.12	.109
	RD ≥2 cm	25	Ref	Ref	Ref	Ref
	NA	6	-	-	-	-
Age at diagnosis	Years		1.03	1.00	1.05	.050

*CI*, confidence interval; *ENR*, extranodal relapse; *HR*, hazard ratio; *ILNR*, isolated lymph node relapse; *RD*, residual disease.

*Hollis et al. Clinical and molecular characterization of ILNR ovarian carcinoma. Am J Obstet Gynecol 2019*.

**Supplementary Table S5:** Multivariable analysis of time to isolated lymph node relapse as a predictor of postrelapse survival in isolated lymph node relapse ovarian carcinoma

Factor	Class	N	HR^multi^	Lower 95% CI	Upper 95% CI	*P*
Relapse type	ILNR	49	0.53	0.33	0.84	.007
	ENR	49	Ref	Ref	Ref	Ref
DFI	≥12 months	46	0.47	0.29	0.75	.006
	<12 months	52	Ref	Ref	Ref	Ref
Age	Years		1.03	1.01	1.06	.006

*CI*, confidence interval; *DFI*, disease-free interval; *HR*, hazard ratio.

*Hollis et al. Clinical and molecular characterization of ILNR ovarian carcinoma. Am J Obstet Gynecol 2019*.

**Supplementary Table S6:** Univariable analyses of specific lymph node site involvement and association with postrelapse survival

Factor	Class	N	HR	Lower 95% CI	Upper 95% CI	*P*
Supraclavicular LN involvement	Yes	6	2.52	0.95	6.69	.064
	No	43	Ref	Ref	Ref	Ref
Pelvic LN involvement	Yes	20	0.73	0.35	1.51	.393
	No	29	Ref	Ref	Ref	Ref
Inguinal LN involvement	Yes	9	0.72	0.28	1.87	.502
	No	40	Ref	Ref	Ref	Ref
Para-aortic LN involvement	Yes	36	1.10	0.48	2.56	.818
	No	13	Ref	Ref	Ref	Ref

*CI*, confidence interval; *HR*, hazard ratio; *LN*, lymph node.

*Hollis et al. Clinical and molecular characterization of ILNR ovarian carcinoma. Am J Obstet Gynecol 2019*.
